# Safety, tolerability, pharmacokinetics and pharmacodynamics of an anti- oncostatin M monoclonal antibody in rheumatoid arthritis: results from phase II randomized, placebo-controlled trials

**DOI:** 10.1186/ar4312

**Published:** 2013-09-24

**Authors:** Ernest H Choy, Marina Bendit, Dana McAleer, Feng Liu, Maria Feeney, Sara Brett, Stefano Zamuner, Andrea Campanile, John Toso

**Affiliations:** 1 Section of Rheumatology, Cardiff University School of Medicine, Tenovus Building, Heath Park, Cardiff, Wales CF14 4XN, UK; 2 Discovery Medicine, Biopharm, GlaxoSmithKline, London, UK; 3 Quantitative Science, GlaxoSmithKline, London, UK; 4 Clinical Pharmacology - Modelling and Simulation, GlaxoSmithKline, London, UK

## Abstract

**Introduction:**

Oncostatin M (OSM) has been implicated in the pathophysiology of rheumatoid arthritis (RA) through its effect on inflammation and joint damage. GSK315234 is a humanised anti-OSM Immunoglobulin G1 (IgG1) monoclonal antibody (mAb). This 3-part study examines the safety, tolerability and efficacy of GSK315234 in patients with active RA.

**Method:**

This was a 3-part (Parts A, B and C), multicenter study. Part A and Part B were randomised, double-blind, placebo-controlled, Bayesian adaptive dose finding studies to investigate the safety, tolerability, efficacy, pharmacokinetics and pharmacodynamics of single (Part A) and 3 repeat (Part B) intravenous infusions of GSK315234 in patients with active RA on a background of methotrexate (MTX). Part C was a single dose, randomised, single-blind, placebo-controlled study to assess subcutaneously administered GSK315234 to patients with active RA on a background of MTX.

**Result:**

The primary endpoint of the study was mean change in DAS28 at Day 28 in Part A and Day 56 in Part B and C. All patients receiving at least one dose of GSK315234 were included in safety analysis. In Part A, there were statistically significant differences in DAS28 between 3 mg/kg and placebo at Day 56, 84 and 91. There was also a statistically significant difference in DAS28 between 0.3 mg/kg, 3 mg/kg and 10 mg/kg, as compared to placebo, at Day 84. Although these changes were small and occurred late, they supported progression to Part B and C to determine the therapeutic potential of GSK315234. For Part B, no significant difference was observed between 6 mg/kg and placebo. For Part C, a statistically significant difference in DAS28 was observed at Day 40, Day 84 and Day 100 between the 500 mg subcutaneous group, as compared to placebo. No significant findings were observed at any of the time points for EULAR response criteria, ACR20, ACR50 or ACR70. An exploratory analysis of clinical, pharmacokinetic and pharmacodynamics data suggests the lack of efficacy may be due to moderate binding affinity and rapid off-rate of GSK315234 as compared to the higher affinity OSM receptor causing a protein carrier effect prolonging the half life of OSM due to accumulation of the OSM/antibody complex in the serum and synovial fluid.

**Conclusion:**

Our data highlighted the importance of binding affinity and off-rate effect of a mAb to fully neutralize the target and how this may influence its efficacy and potentially worsen disease activity. Using an anti-OSM mAb with high affinity should test this hypothesis and examine the potential of OSM as a therapeutic target in RA.

**Trial registration:**

ClinicalTrials.gov no: NCT00674635

## Introduction

Rheumatoid arthritis (RA) is characterized by chronic inflammation and destruction of articular joints. Joint damage leads to physical disability. Despite recent advances in the treatment of RA with early use of methotrexate (MTX), a combination of disease modifying anti-rheumatic drugs (DMARDs) and the introduction of biologics, fewer than 50% of patients achieved disease remission [1]. Consequently, the majority of patients continue to suffer from active disease. As a result, there is a need for new treatments to address this ongoing burden of disease.

Cytokines have a major role in causing joint damage. Oncostatin M (OSM) is a member of the interleukin (IL)-6 family of secreted cytokines and is present in the inflamed synovium and blood of patients with RA [2,3]. It is a pleiotropic cytokine with diverse biological functions relevant to all the major aspects of the pathogenesis of RA. These include activation of endothelium and fibroblasts, stimulation of the inflammatory mediator release and proliferation of synovial cells, promotion of angiogenesis, induction of cartilage breakdown and osteoclastogenesis leading to bone erosion [4-8]. In animal models of RA, anti-OSM antibody ameliorated disease activity [9].

GSK315234 is a humanised anti-OSM immunoglobulin G1 (IgG1) monoclonal antibody (mAb), which was developed for the treatment of RA. GSK315234 recognises and functionally blocks an epitope in the Site II region of the OSM molecule, preventing its interaction with the cell surface signaling receptor gp130 and consequently all the biological functions of OSM. Administration of GSK315234 to patients with active RA was expected to reduce the signs and symptoms of RA due to the inflammatory effects of OSM, reduce pannus formation and synovial cellular infiltrate due to inhibition of synovial cell proliferation and reduction in angiogenesis and reduce joint damage due to the destructive effects of OSM on cartilage and bone.

The aim of this clinical study was to investigate the safety, tolerability, pharmacokinetics and pharmacodynamics of GSK315234 in RA using Bayesian adaptive clinical trial design. Traditional parallel group clinical trial design requires the sample size to be predetermined and assessments completed in all subjects before data are analysed. The design is inefficient for phase II dose escalating trials in which low dose treatment groups are unlikely to show efficacy but many patients have to be recruited into these groups. Bayesian adaptive clinical trial design was developed from sequential designs in which the design can be changed based on knowledge gained from interim analyses. Adaptive designs allow trials to start out with a small up-front commitment of sample size and then extend them if necessary. Such adaptive trial designs can make a range of protocol changes, including changing the sample size or randomisation fraction and dropping or adding treatment arm(s). Changing treatment schedules and sample sizes allows the trial to be adjusted to maximize efficiency of the trial; historically this created very large demand on computation, but modern computer hardware and software have made this feasible.

## Methods

This was a three-part (Parts A, B and C), multicentre study to investigate the safety, tolerability, efficacy, pharmacokinetics and pharmacodynamics of intravenous (IV) GSK315234 in Parts A and B and subcutaneous (SC) GSK315234 in Part C in patients with RA. Parts A and B were randomised, double-blind, placebo-controlled, Bayesian adaptive dose-finding studies to investigate the effect of single (Part A) and three repeat (Part B) IV infusions of GSK315234 in patients with active RA on a background of MTX. Part C was a single dose, randomised, single-blind, placebo-controlled study of SC administered GSK315234 in patients with active RA on a background of MTX.

### Patients

Patients between 18 and 75 years of age, who fulfill 1987 American College of Rheumatology (ACR) classification criteria of RA were recruited [10]. They must have had active disease: Disease Activity Score 28 (DAS28) of >4.2 at screening and a pre-dose C-reactive protein (CRP) level of ≥0.5 mg/dl or an erythrocyte sedimentation rate (ESR) level ≥28 mm/hour at screening and pre-dose. Patients should have received at least three months of MTX and have been on a stable dose (up to 25 mg/week) for at least eight weeks prior to screening and be willing to remain on this dose throughout the study. Concomitant sulfasalazine or anti-malarial was permissible if it was taken in addition to MTX, and the dose was stable for at least four weeks for sulfasalazine and three months for anti-malarial prior to screening. Other DMARDs must have been withdrawn for more than one month prior to screening. Other oral anti-rheumatic therapies, such as non-steroidal anti-inflammatory drugs (NSAIDs), COX-2 inhibitors, oral glucocorticoids, were permitted providing the dose is ≤10mg/day of prednisolone (or equivalent) and stable for at least four weeks prior to screening and remains unchanged through the study. Patients must use acceptable contraception during the course of the study. Patients were excluded if they had received prior biologic therapy, have active infection, previous exposure or past infection caused by *Mycobacterium tuberculosis*, positive Hepatitis B surface antigen or Hepatitis C antibody result at screening, history of HIV or other immunodeficiency disease, history of malignancy, except for adequately treated non-invasive cancers of the skin (basal or squamous cell) or carcinoma *in situ* of the uterine cervix, positive pregnancy test, elevated liver function tests on more than one occasion: transaminases or alkaline phosphatase >3 times the upper limit of normal (ULN) or total bilirubin >1.5 times ULN, or any significant medical conditions. Patients with haemoglobin (Hb) <10 g/dl or platelet count <150 × 10^9^/l were also excluded.

The study was approved by Institution Review Boards (Serbia: Institute of Rhematology Belgrade, Institute for Prevention and Treatment and Rehabilitation of Rheumatic and Cardiovascular Diseases Belgrade; Ukraine: Zaporizhzhya State Medical University, Keiv National Medical University, National Scientific Center, Institute Kiev, Keiv National Medical University, Donetsk State Medical University, Lviv National Medical University; New Zealand: Waikato Hospital, Hamilton, Christchurch Clinical Studies Trust Christchurch, P3 Research Wellington; Russia: State Research Institute Novosibirsk, Yaroslavi Regional Clinical Hospital, Yaroslavi; Smolensk Regional Clinical Hospital, Moscow Regional Research Clinical Institute Rhematology Institute Moscow; Australia: Nucleus Network Melbourne, Austin Center for Clinical Studies Heidelberg, Princess Alexandra Hospital Wooloongabba) and Charing Cross Research Ethics Committee, UK and registered (ClinicalTrials.gov no: NCT00674635). All patients gave signed informed consent. The study was conducted in accordance with the guiding principles of the Declaration of Helsinki.

### Dosing and study design

In Part A, initially, six cohorts of eight patients each were enrolled (Cohorts 1 through 6). After the first interim analysis, an additional two cohorts of patients were enrolled (Cohort 7 and Cohort 8). Eligible patients within each cohort were randomized to GSK315234 (n = 6) or placebo (n = 2). A starting dose of 0.03 mg/kg GSK315234 was identified, and a Bayesian adaptive dose-finding algorithm based on a measure of clinical response (weighted mean DAS28) on Day 14 post-dose was used to identify subsequent doses that provided 90% of maximal benefit based on trial simulation of the Bayesian adaptive pharmacokinetics and pharmacodynamics (PK/PD) design. Patients in Cohorts 1 through 6 received 0.03 mg/kg, 0.3 mg/kg, 3 mg/kg (2 cohorts of patients were enrolled at this dose level), 10 mg/kg and 30 mg/kg of GSK315234; doses were administered in a dose rising fashion. Cohorts 2 through 6 were dosed a minimum of three weeks after dosing of the last patient in the previous cohort. Cohorts 7 and 8 enrolled simultaneously, and patients received 10 mg/kg or 20 mg/kg GSK315234.

Part B was a randomized, double-blind, placebo-controlled, repeat dose study based on changes in DAS28 and PK in Part A. Prior to administration of the first dose, eligible patients (n = 54) were randomized in a 2:1 ratio to receive GSK315234 (n = 37) or placebo (n = 17). For each patient, doses were administered approximately four weeks apart.

In Parts A and B, GSK315234 or placebo was administered by slow IV infusion over two hours.

Part C was a randomized, single-blind, placebo-controlled, single SC dose study. Eligible patients (n = 17) were randomized on a 3:1 basis to GSK315234A (n = 12) or placebo (n = 5). One patient in the placebo arm was randomized and dosed but was withdrawn as the DAS28 score was lower than 4.2 at pre-dose on day 1. Patients were administered 500 mg of GSK315234 or matching placebo as five SC injections of 1 mL each (multiple injections were needed to administer the full dose). SC injections were administered on the abdomen, rotating sites around the umbilicus.

A central randomization schedule generated using the GSK Randall system was used in all parts of the study. There is no stratification of sites or countries. GSK315234 or placebo was administered in a blinded fashion so that both patients and investigators remained blinded to treatment allocation.

### Assessments

The primary efficacy endpoint was the DAS28 [11] response rate on Day 28 in Part A and Day 56 in Parts B and C. Secondary endpoints were ACR [12] and European League Against Rheumatism (EULAR) response rates [13], together with Outcome Measure in Rheumatology (OMERACT) core component measures [14]: tender/painful count; swollen joint count; Patient’s Assessment of Arthritis Pain (100 mm visual analogue scale (VAS)); Patient’s and Physician’s Global Assessments of Arthritis (100 mm VAS); Health Assessment Questionnaire-Disability Index (HAQ-DI) [15] and Multi-dimensional Assessment of Fatigue (MAF). Laboratory efficacy measures included CRP and ESR.

Safety assessments including adverse events (AEs), vital signs, electrocardiograms and clinical laboratory tests (haematology, biochemistry and urinalysis) were carried out at each study visit.

### Pharmacokinetics and pharmacodynamics

PD biomarkers after single and repeat IV doses included, but were not limited to, serum OSM and GSK315234A-OSM complexes. Immunogenicity was measured by human anti-GSK315234A antibodies.

### Sample size estimation and sensitivity

In Part A, the use of a non-linear mixed effects procedure required simulation techniques to estimate power for a given sample size and expected magnitude of effect. Trial simulations of the Bayesian adaptive PK/PD design using a nonlinear PK/PD model were conducted using typical parameter estimates that six cohorts of eight patients each would provide power in excess of 95% to detect a PK/PD maximum effect value of 66% inhibition from baseline. The probability of a false positive under the null hypothesis was approximately 5%. When the number of cohorts was increased to eight, the power increased marginally accompanied by a steeper cumulative distribution curve. When the magnitude of response was 33%, the power reduced to 80%. If the response to GSK315234 had a slower onset but similar sized response to adalimumab, the power for the PK/PD analysis was 80%. Overall, a sample size of 48 (six cohorts) or 64 (eight cohorts) would provide at least 80% power assuming a similar response to adalimumab.

In Part B, a maximum of 54 patients was planned for enrollment. A treatment difference of 0.95 between the selected dose and placebo in DAS28 scores 56 days post dose could be detected with approximately 90% power based on preliminary estimates of between subject variability of DAS28 scores seen in the interim analysis of Part A. This assumes a standard deviation of 1.15 in the GSK315234 dose group and 1.25 in the placebo dose group, a two-sided test and an overall alpha of 5%.

In Part C, no statistical techniques were used to determine the sample size.

### Statistical analysis plan

A repeated measure analysis using a mixed effect model was used, including treatment, visit, and treatment by visit interaction as fixed effects and patient as a random effect to analyse the primary efficacy endpoint (mean change from baseline in DAS28 scores at Day 28). Other effects such as baseline, baseline by visit, country, gender, age and baseline OSM level were fitted into the model when deemed necessary.

A Bayesian normal dynamic linear model was applied to DAS28 data of evaluable patients to estimate the dose–response relationship at Day 28, Day 56, and Day 84 in Part A.

Descriptive statistical methods were used to summarize all primary and secondary efficacy variables (absolute value and change from baseline). Significance tests were carried out at the 2-sided 5% level. Changes from baseline in tender joint count, swollen joint count, patient’s pain assessment, patient’s and physician’s global assessment, CRP, ESR, HAQ-DI and MAF were compared between treatment groups. If the assumption of normality was not satisfied for these data, then the data were transformed prior to analysis.

### Weighted mean DAS28 score

The weighted DAS28 was defined by the area under the curve (AUC) divided by the number of days. The weighted mean DAS28 was calculated and summary statistics are presented for Parts A, B and C. The difference in the mean of the two treatment groups is presented together with the corresponding 2-sided 95% confidence interval and the 2-sided *P*-value.

For EULAR, ACR20, ACR50 and ACR70 response rates, the Cochran Mantel Haenszel (CMH) test was used for comparing the responder rates at each visit for GSK315234A versus placebo stratified by subgroup factors.

## Results

**Figure 1:**
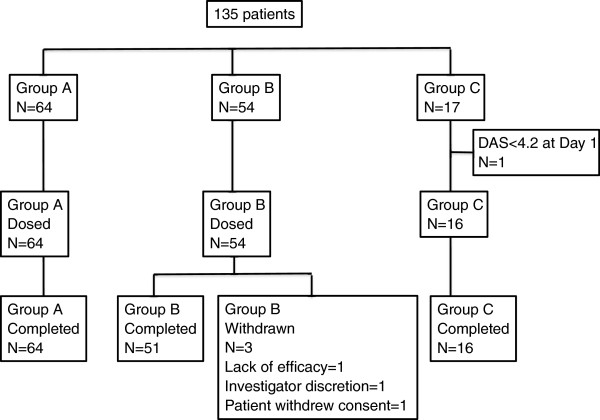
Patient disposition.

In total, 135 patients with RA were dosed (64 in Part A, 54 in Part B and 17 in Part C). Their demographic details are given in Table 1. All patients in Part A completed the study. Three patients in Part B withdrew due to lack of efficacy, investigator discretion and patient withdrawing consent. One patient in Part C who received one dose of treatment had to be withdrawn from the study and analysis due to protocol violation (Figure 1).

**Table 1:** Demographic details of the patients

	**Part A**							**Part B**	**Part C**	**Pooled**
	**0.03 mg/kg**	**0.06 mg/kg**	**0.3 mg/kg**	**3 mg/kg**	**10 mg/kg**	**20 mg/kg**	**30 mg/kg**	**6 mg/kg**	**500 mg**	**Placebo**
Number of patients randomized	3	1	8	12	12	6	6	37	12	38^a^
Age in Years, Mean (SD)	45.7 (9.3)	46.0	50.3 (3.3)	51.1 (8.0)	52.8 (13.4)	58.8 (8.3)	53.2 (5.0)	53.7 (10.1)	55.6 (13.2)	56.2 (11.4)
Gender, n (%)										
Female: (%)	3 (100)	1 (100)	5 (63)	9 (75)	5 (42)	4 (67)	4 (7)	23 (62)	10 (83)	35 (92)
Male: (%)			3 (37)	3 (25)	7 (58)	2 (33)	2 (33)	14 (38)	2 (17)	3 (8)
BMI in kg/m^b^**,** Mean (SD)	28.0 (3.6)	25.7	27.1 (3.9)	24.8(3.1)	25.2(2.8)	28.3 (3.9)	25.3 (5.8)	26.4 (3.5)	27.9(5.1)	27.5 (4.4)
Disease Duration in years, Mean (SD)	8.4 (10.2)	2.5	6.7 (7.0)	6.0 (6.6)	3.92 (3.4)	14.7 (10.4)	13.4 (13.3)	8.0 (6.9)	8.92 (7.8)	8.0 (7.7)
Number of Prior DMARDs, Mean (SD)	3 (0)	2	2.4 (0.5)	2.7 (0.8)	2.5 (0.7)	3.3 (1.5)	2.7 (1.2)	2.4 (0.7)	2.2 (0.6)	2.4 (0.7)
Rheumatoid Factor Positive N (%)	2 (66)	1 (100)	8 (100)	10 (83)	7 (58)	6 (100)	6 (100)	31 (84)	12 (100)	31 (82)
Anti-CCP antibodies positive N (%)	2^2^(67)	1 (100)	8 (100)	9 (75)	10 (83)	6 (100)	6 (100)	NA	NA	12 (75)
Baseline DAS28ESR, Median	5.87	6.77	6.47	6.66	6.15	6.91	6.59	6.43	6.40	6.19

^a^One patient in the placebo group was randomized and dosed but excluded from the analysis of efficacy because of ineligible DAS28 score (<4.2) pre-dose on day 1; ^b^Data missing from 1 patient. BMI, body mass index: DAS28, Disease Activity Score 28; DMARDs, disease modifying anti-rheumatic drugs, SD, standard deviation.

### Efficacy

**Figure 2:**
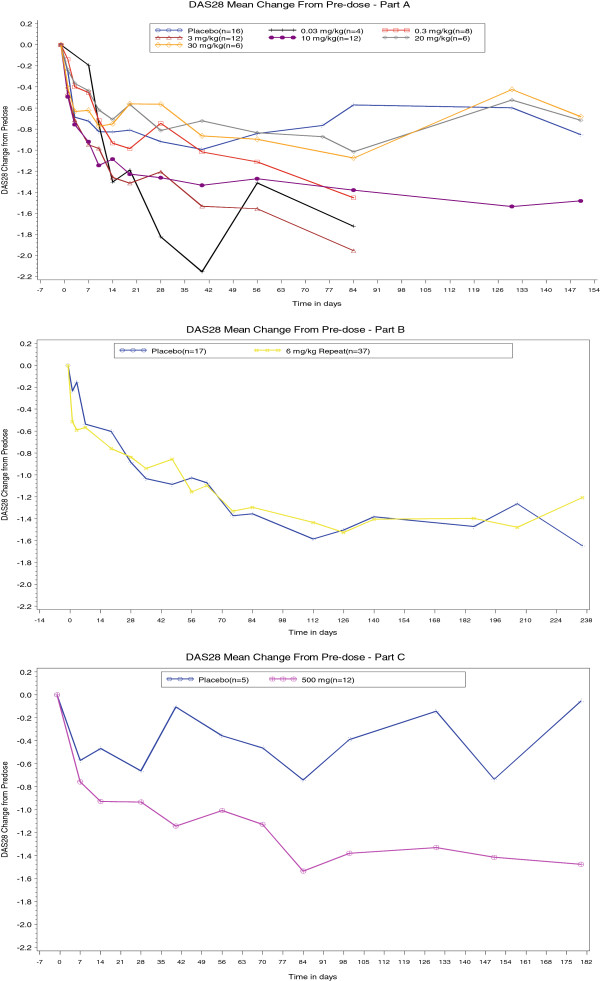
Mean changes in Disease Activity Score 28 (DAS28) in Parts A, B and C.

In Part A, there was a statistically significant difference in DAS28 between 3 mg/kg and placebo (adjusted mean difference was −0.71, *P* <0.05) at Days 56, 84 and 91. There was a statistically significant difference between 0.3 mg/kg, 3 mg/kg and 10 mg/kg compared to placebo, at Day 84. The largest adjusted mean change in DAS28 from baseline was observed for the 3 mg/kg group at Day 84 (adjusted mean change = −1.95 (*P* <0.05) and the difference, compared to placebo, was −1.43 (*P* <0.05). For Part B, no significant difference was observed between 6 mg/kg and placebo. For Part C, a statistically significant difference was observed at Days 40, 84 and 100 between the 500 mg SC group compared to placebo. Figure 2 shows the changes in DAS28 with time in parts A, B and C.

No significant findings were observed at any of the time points for EULAR response criteria, DAS28 remission, ACR20, ACR50 or ACR70.

For swollen joint count, a statistically significant benefit for the comparison of GSK315234 versus placebo was observed for the 0.3 mg/kg, 3 mg/kg and 10 mg/kg dose groups at various time points in Part A. There was no statistically significant improvement of 6 mg/kg over placebo at any time point in Part B.

No statistically significant improvement in HAQ-DI compared to placebo was observed at any time point for all treatment groups (Parts A, B and C).

For both ESR and CRP, there was no statistically significant improvement with GSK315234 compared to placebo at any time point after the 24 hour time-point in Parts A, B and C.

For the global fatigue index measured by the MAF, there was a statistically significant improvement in the 10 mg/kg dose group in Part A compared to placebo at Day 7 (*P* = 0.0216). There were no other statistically significant changes in GSK315234 dose groups compared to placebo for any other time point for all treatment groups.

### Safety

Single and repeat doses of GSK315234 were generally well tolerated. AEs were reported in 48% of the patients administered GSK315234 (in any part and dose level) and 32% of the pooled placebo patients (Table 2). No AEs led to patient withdrawal from the study. Overall, the most commonly reported AEs were worsening of RA, increase in alanine transferase, pyrexia, headache, blood pressure increase and diarrhea. All AEs were Grade 1, 2 or 3; there were no subjects with Grade 4 or higher AEs.

**Table 2:** Total number of patients (%) with any adverse event and decrease in platelet

		**Adverse events number of patients (%)**		**Percentage decrease in platelet number of patients (%)**			
**Part**	**Treatment group (N)**	**Any AE**	**Any AE (days 1–28)**	**25 to 38%**	**39 to 51%**	**52 to 85%**	**All**
A	0.03/0.06 mg/kg IV (N = 4)^a^	2 (50)	1 (25)	0	0	0	0
	0.3 mg/kg IV (N = 8)^a^	2 (25)	2 (25)	1 (13%)	0	0	1 (13%)
	3 mg/kg IV (N = 12)	7 (58)	5 (42)	6 (50%)	0	0	6 (50%)
	10 mg/kg IV (N = 12)	6 (50)	5 (42)	4 (33%)	0	1 (8%)	5 (42%)
	20 mg/kg IV (N = 6)	5 (83)	4 (67)	2 (33%)	2 (33%)	0	4 (67%)
	30 mg/kg IV (N = 6)	4 (67)	3 (50)	1 (17%)	2 (33%)	0	3 (50%)
B	6 mg/kg repeat IV (N = 37)	15 (41)	8 (22)	10 (27%)	4 (11%)	1 (3%)	15 (41%)
C	500 mg SC (N = 12)	6 (50)	6 (50)	5 (42%)	2 (17%)	0	7 (58%)
Pooled (Parts A, B, C)	All Placebo (N = 38)	12 (32)	8 (21)	8 (21%)	1 (3%)	1 (3%)	10 (26%)

AE, adverse event; IV, intravenous patient; SC, subcutaneous patient.

There was no infusion reaction. SC injections of GSK315234 did not cause any injection site related AEs.

There were more treatment-related AEs in the GSK315234 treated patients (16/64, 25%) compared to the placebo treated patients (0, 0%).

Non-fatal severe adverse events (SAEs) were reported for two patients: breast cancer and acute sinusitis.

**Figure 3:**
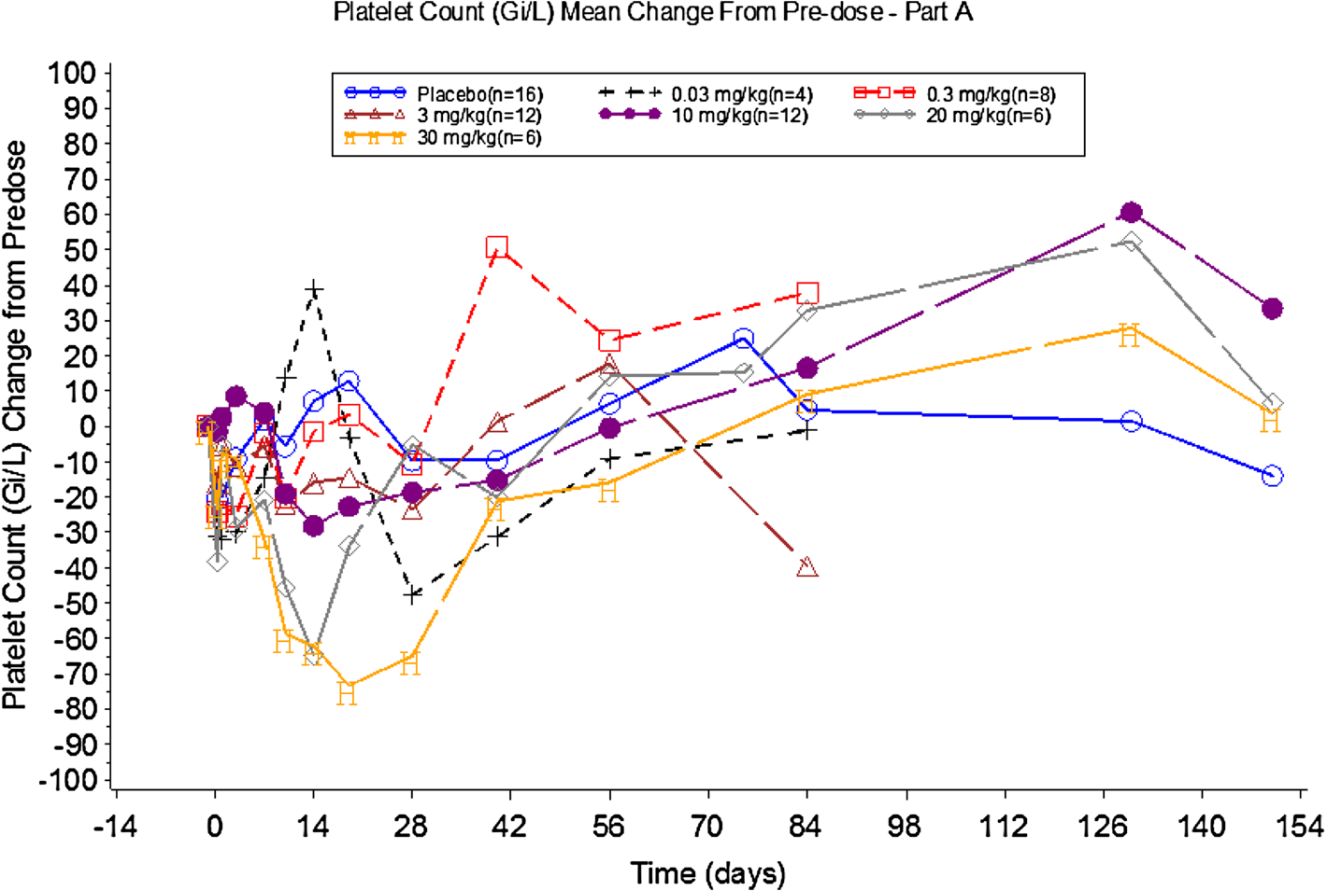
Mean percentage change in platelet count before and after treatment in Part A.

There was a dose related decrease in platelet number, although all platelet counts were within the normal reference range. Percentage and mean changes from baseline are detailed in Table 2 and Figure 3, respectively. These reductions generally occurred around Day 19, appeared to be dose dependent and resolved within two to four weeks. This decrease in platelet count is consistent with the pharmacology of GSK315234 and appeared to be dose proportional with platelets demonstrating a greater decrease from baseline over a longer period of time.

### Immunogenicity

Anti-GSK315234 antibodies were observed in about 25% of patients at all dose levels except for the 0.3 mg/kg and 20 mg/kg in which no anti-GSK315234 antibodies were observed. Treatment failure did not relate to the presence anti-GSK315234.

### Pharmacokinetics and pharmacodynamics

**Figure 4:**
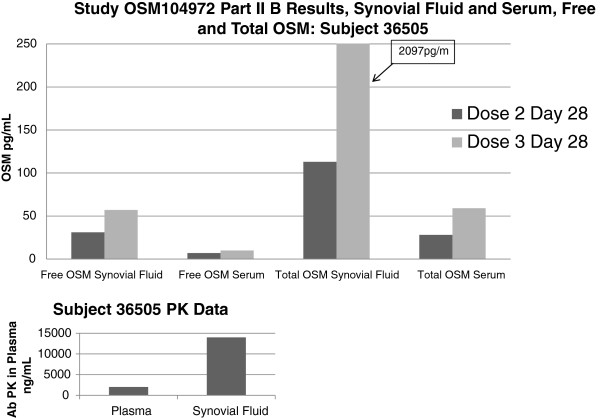
Free and Total oncostatin M in one patient following repeating dosing with GSK315234.

After single and multiple IV infusion dosing, GSK315234 peak concentrations were achieved on average at two to four hours after dosing. Following the peak, concentrations declined slowly with a mean terminal t½ of about 300 to 400 hours after both single and repeat dosing. No accumulation was observed after multiple IV infusions in Part B, with the mean accumulation ratio being 1.08. An exploratory PK/PD analysis showed some improvement in DAS28 at lower exposures (doses ≤3 mg/kg), supported by a significant effect on CRP and IL-6 (U-shape exposure-response). One potential explanation for this data is moderate to poor binding affinity (800 pM) and rapid off rate of GSK315234 compared to the higher affinity OSM receptor (150 pM) causing a protein carrier effect. This is supported by synovial fluid data in which, although the majority of the OSM is complexed, there was a significant amount of free OSM present in the synovial fluid (Figure 4).

## Discussion

Administration of GSK315234 to patients with active RA was expected to reduce signs and symptoms of RA due to the effects of OSM on inflammation, pannus formation, synovial cellular infiltrate and joint damage. The results of this three-part, multicenter study show no evidence of efficacy in the repeat dose, powered portion of the study. However, there is evidence of pharmacology and downstream biologic effect with a dose dependent decrease in platelets. The platelet reduction is not considered clinically significant or a safety related issue since in all patients, platelet counts remained within the normal range. OSM stimulates hepatocytes to release acute phase reactants [16-18]. Anti-OSM mAb does not bind to IL6; therefore, GSK315234 does not have a direct effect on the acute phase response. Any change in ESR or CRP would be due to inhibition of OSM and indirectly through reduction in disease activity.

In Part A, the single dose study showed a statistically significant clinical response in the 3 mg/kg group on Days 56 and 84 and the 10 mg/kg group on Day 84. One potential explanation is that OSM is not a good therapeutic target in RA and the positive results in the single dose study were false positives. Alternatively, if the observations that statistically significant changes in DAS28 compared to placebo occurred in the 3 mg/kg group on Days 56 and 84 and in the 10 mg/kg group on Day 84, yet the effect on DAS28 scores at high doses (20 and 30 mg/kg) appeared to be worse compared with the low-medium doses are true, it would suggest a U-shape response curve. Such a response has been observed in some medical conditions but not in clinical trials of musculoskeletal diseases [19]. Inadequate neutralisation of other cytokines due to a protein carrier effect has been reported in the literature and included situations in which there is a high target load, for example, IL-6 in cancer patients [20]. We conducted an exploratory PK/PD analysis which showed some improvement in DAS28 at lower exposures (doses ≤3 mg/kg), supported by a significant effect in CRP and IL-6 (U-shape exposure-response). One potential explanation is for a protein carrier effect due to the moderate to poor binding affinity (2.14 nM at 37°C by BiaCore) and rapid off-rate (1.73× 10^3^) of GSK315234 compared to the higher affinity OSM receptor (150 pM) at the site of action in the synovial joint. We hypothesize that GSK315234 binds to circulating OSM. At low doses of GSK315234, it was unable to neutralise OSM. At a moderate dose, GSK315234 effectively neutralised the activity of OSM. At high doses of GSK315234, all OSM will be complexed to GSK315234. The GSK315234-OSM complex will have a longer half-life than OSM. However, due to the high off rate and low binding affinity of GSK315234 relative to the OSM receptor, GSK315234 acts as a carrier of OSM and OSM is released in the synovial joint. Our results suggest that other factors, such as accumulation of the OSM complex and rate of dissociation from GSK315234, need to be properly considered (Figure 3).

Nevertheless, both single and repeat dosing with GSK315234 was generally well tolerated. The PK/PD analysis suggested that moderate affinity and rapid off rate of GSK315234 may lead to a U-shape dose response. A higher affinity anti-OSM antibody is needed to truly examine the role of OSM in RA. It may also have the potential to treat fibrotic lung disease based on OSM biology [21].

## Conclusion

Our data highlight the importance of binding affinity and off-rate effect of a mAb to fully neutralize the target and how this may influence its efficacy and potentially worsen disease activity. Using an anti-OSM mAb with high affinity should test this hypothesis and examine the potential of OSM as a therapeutic target in RA.

## Abbreviations

ACR: American College of Rheumatology; AEs: adverse events; CMH: Cochran Mantel Haenszel; CRP: C-reactive protein; DAS28: Disease Activity Score 28; DMARDs: disease modifying anti-rheumatic drugs; ESR: erythrocyte sedimentation rate; EULAR: European League Against Rheumatism; IgG1: Immunoglobulin G1; IL: interleukin; IV: intravenous; HAQ-DI: Health Assessment Questionnaire Disability Index; Hb: haemoglobin; MAF: Multi-dimensional Assessment of Fatigue; mAb: monoclonal antibody; MTX: methotrexate; NSAIDs: non- steroidal anti-inflammatory drugs; OMERACT: Outcome Measure in Rheumatology; OSM: Oncostatin M; PK/PD: pharmac kinetics and pharmacodynamics; RA: Rheumatoid arthritis; SC: subcutaneous; ULN: upper limit of normal; VAS: visual analogue scale.

## Competing interests

Ernest Choy has served as a consultant and on the advisory board of GSK. Marina Bendit, Dana McAleer, Feng Liu, Maria Feeney, Sara Brett, Stefano Zamuner, Andrea Campanile, John Toso are employees of GSK.

## Authors’ contributions

EHC drafted the manuscript, contributed to study design, conduct of the study and analysis and interpretation of the result. JT contributed to the manuscript, study design, analysis and interpretation of the results and participated in the conduct of the study. SZ contributed to the manuscript, analysis and interpretation of the results. SB contributed to the manuscript, analysis and interpretation of biomarker results. FL contributed to the manuscript, data analysis and interpretation of the results and participated in the conduct of the study. DM contributed to the manuscript, study design, analysis and interpretation of the results and participated in the conduct of the study. MB contributed to the manuscript, study design, acquisition and analysis of the data and participated in the conduct of the study. MF contributed to the manuscript, analysis and interpretation of biomarker results. AC contributed to the manuscript, study design, analysis and interpretation of the results and participated in the conduct of the study. All authors read and approved the final manuscript.
